# Lower Healthcare Access and Its Association With Individual Factors and Health-Related Quality of Life in Adults With Rare Diseases in Switzerland

**DOI:** 10.3389/ijph.2024.1607548

**Published:** 2024-09-25

**Authors:** Susanne Wehrli, Andrew A. Dwyer, Matthias R. Baumgartner, Carmen Lehmann, Markus A. Landolt

**Affiliations:** ^1^ Division of Child and Adolescent Health Psychology, Department of Psychology, University of Zurich, Zurich, Switzerland; ^2^ Children’s Research Center, University Children’s Hospital Zurich, Zurich, Switzerland; ^3^ University Children’s Hospital Zurich, Zurich, Switzerland; ^4^ Connell School of Nursing, Boston College, Chestnut Hill, MA, United States; ^5^ Massachusetts General Hospital, Harvard Medical School, Boston, MA, United States; ^6^ Department of Metabolism, University Children’s Hospital Zurich, Zurich, Switzerland

**Keywords:** healthcare access, health outcomes, rare disease, health-related quality of life, quality of life

## Abstract

**Objectives:**

This study aims to determine clusters of access to healthcare among adults with rare diseases in Switzerland, identify associated individual characteristics of access, and impact on health-related quality of life (HRQoL).

**Methods:**

Swiss adults (*N* = 341) diagnosed with a rare disease completed an online survey including the Perception of Access to Healthcare Questionnaire (PAHQ) and Short Form Health Survey (SF-12). We employed partition around medoids algorithm to identify patient clusters based on the PAHQ. Various sociodemographic/disease-related factors and HRQoL were assessed.

**Results:**

We identified two patient clusters: higher (*n* = 227) and lower access (*n* = 114). Significantly associated with lower access were an unstable disease course (*p* < 0.05), increased number of misdiagnoses (*p* < 0.05), and diseases affecting the nervous system (*p* < 0.01). Membership in the lower access cluster was significantly associated with worse HRQoL (*p* < 0.05).

**Conclusion:**

Findings highlight the need for comprehensive assessment of healthcare access in adults with rare diseases and identifies potential targets for tailored interventions.

## Introduction

Rare diseases encompass approximately 7,000 different conditions affecting between 263 and 446 million people worldwide (i.e., 3.5%–5.9% of the global population) [1, 2]. Rare diseases vary in terms of disability, disease progression, and life expectancy, ranging from diseases that are life altering yet manageable (e.g., phenylketonuria) to unstable, progressive diseases with reduced life expectancy (e.g., Duchenne muscular dystrophy) [3, 4]. Early detection and intervention can mitigate disability in some cases, yet many patients face substantial barriers to healthcare access. Challenges include healthcare professionals’ limited knowledge, lengthy diagnostic process (i.e., “diagnostic odyssey”), and frequent misdiagnoses [2] that impact individual health outcomes and place considerable strain on healthcare systems [5].

### Access to Healthcare in the Context of Rare Diseases

Access to healthcare is a complex, multidimensional construct that varies widely across scientific contexts [6, 7]. The complexity is especially pronounced for rare diseases, as the interplay of health system characteristics, providers, and patients all affect access. The Penchansky and Thomas framework [8–10] delineates access across six critical dimensions: accessibility, availability, acceptability, affordability, adequacy, and awareness, that are essential for aligning services with user needs. The Perception of Access to Healthcare Questionnaire (PAHQ) is based on the framework and includes subscales for each dimension [11].

Switzerland’s healthcare system is characterized by a decentralized structure, with healthcare services provided through a mix of public and private sectors [12]. All residents are required to have mandatory health insurance, which ensures access to basic healthcare services, including those for chronic and rare diseases. Rare disease patients in Switzerland face significant challenges, similar to those seen across the EU. Diagnostic delays and frequent misdiagnoses are major concerns, often due to limited experience and knowledge among healthcare professionals, particularly in primary care [13]. This lack of rare disease knowledge among healthcare providers, leads patients to turn to the internet, sometimes relying on unreliable sources of information [14, 15]. Access to specialized treatments is another issue, as some therapies are unavailable or difficult to access within the country, leading patients to seek care abroad [13], this is particularly difficult for individuals with impaired mobility [14] thus creating issues in accessibility and availability. Also, patients and families are frequently faced with extended wait times for specialist appointments and must navigate disjointed, uncoordinated care [16, 17]. Insurance coverage for costly orphan drugs is inconsistent, and administrative barriers add further complications regarding the affordability dimension [18]. Financial strain may be further exacerbated as rare diseases limit employment opportunities [14, 19]. To address these issues, Switzerland has implemented initiatives such as the Swiss National Concept for Rare Diseases (NKSK) and the National Coordination for Rare Diseases (KOSEK). These programs aim to improve diagnosis, care structures, and coordination of expertise through specialized centers [20]. Such multifaceted challenges highlight the unique difficulties that individuals with rare diseases face in accessing healthcare within Switzerland across the Penchansky and Thomas dimensions [8].

### Associated Individual Characteristics of Healthcare Access and the Link With Health-Related Quality of Life

The Penchansky and Thomas model [8] neither incorporates individual-level characteristics such as predisposing variables (e.g., gender, age, marital status, socioeconomic status, etc.), nor enabling factors (e.g., insurance status, employment status, etc.) and need factors (e.g., psychiatric comorbidities, chronic conditions, etc.). Such factors are identified in other models, namely, the Andersen model [21]. Individual, enabling, and need factors are typically associated with healthcare utilization [6, 21]. Recent studies have begun to integrate features from the Andersen model into the framework of Penchansky and Thomas [8] to create a more comprehensive approach that combines both systemic access dimensions and individual patient characteristics [22, 23].

The Andersen model [21] is salient for rare diseases yet remains relatively underexplored to date. Research indicates critical access variables including misdiagnosis, that undermines trust and impedes proper care [24, 25], and unstable disease course that complicate providing adequate care [19]. Conversely, a prolonged time post-diagnosis enhances patients’ ability to navigate healthcare services. Moreover, patient organizations play a crucial role in empowering patients to navigate health systems, inform research agendas, and shape policy [26]. Thus, factors like misdiagnosis, disease stability, diagnostic timeliness, and patient organization involvement are vital in determining healthcare access for individuals with rare diseases.

Healthcare access, health system efficiency, and key health outcomes, including health-related quality of life (HRQoL) and life expectancy, are all linked playing critical roles in both healthcare system performance and individual wellbeing [27, 28]. Individuals with rare diseases often have lower HRQoL compared to healthy controls highlighting the adverse impact of inadequate healthcare access [29, 30]. To date, the specific relationship between healthcare access and HRQoL among individuals with rare diseases remains under-researched and poorly elucidated.

### Objectives

Previous rare disease research has neither fully explored the multidimensional nature of access nor the relationship between access, its associated individual characteristics as proposed by the Andersen model [17], and HRQoL. We posit that such insights will inform development of targeted interventions to bridge gaps in care experienced by adults with rare diseases. We employ exploratory machine learning techniques and sought to avoid formulating specific hypotheses to reduce the risk of overfitting, which is a significant concern for reproducibility [31].

First, we analyze healthcare access across all dimensions within the Swiss healthcare system using partition around medoids (PAM) algorithm. The analysis aimed to identify clusters representing different levels of healthcare access, characterized by unique combinations of subscale scores within the framework established by Penchansky and Thomas [8]. Second, we explore individual characteristics associated with healthcare access for individuals with rare diseases. Rather than categorizing these characteristics according to the Anderson model [21] (i.e., predisposing, enabling, and need factors), we acknowledge the complex interrelationship and incorporate all characteristics in the same analysis. Last, we investigate how membership in specific access clusters is associated with physical and mental HRQoL to gauge the effects of limited healthcare access on patient wellbeing and validate the significance of identified access clusters.

## Methods

### Study Design and Data Collection

We conducted an anonymized, online, cross-sectional survey of German, French, English, or Italian speaking Swiss adults with rare diseases. Participants were enrolled in collaboration with patient organizations and physicians affiliated with the University Children’s Hospitals of Zurich, Bern, and Lausanne, respectively. Participants were recruited through newsletters, social media platforms, physician emails, and email blasts to membership of patient advocacy groups. Participants received no financial compensation for participation and participants provided opt-in electronic informed consent prior to starting the online survey. Included participants were adults (18+ years.); residing in Switzerland; proficient in either German, French, Italian, or English; and had a medically confirmed rare disease diagnosis – i.e., condition affecting <5 in 10,000 people per the European Parliament and Council Regulation on Orphan Medicinal Products [32].

A total of 606 individuals participated. Several participants were excluded from the analysis for not providing consent (*n* = 4), not residing in Switzerland (*n* = 4), unable to identify the name of their disease (*n* = 5), and not meeting rare disease definition criteria (*n* = 15). The rare disease criteria was validated using orphanet prevalence estimates. An additional 229 were excluded for not completing the PAHQ. In total, 39 participants were retained despite incomplete SF-12 data - as no significant differences were found in their PAHQ subscale scores (Supplementary Table S1). Mann-Whitney U tests were performed, due to a non-normal distribution of the data according to the Shapiro-Wilk test.

To preserve variance in this heterogeneous sample, outliers identified via boxplot analysis (Supplementary Figure S2) were not removed. No outliers were detected among SF-12 summary scores (Supplementary Figure S3). Twenty two diagnoses were identified in the International Classification of Diseases 10th Revision (ICD-10) [33] but not the 11th Revision (ICD-11) [34] (Supplementary Table S4). Participants with disease types reported in fewer than ten cases were excluded as variables, but not excluded as cases from the analyses, to avoid inaccuracies due to low event ratios [35]. The final sample size included 341 participants.

### Measures

#### Individual Characteristics

Participants provided sociodemographic information including sex, age, nationality, medical insurance coverage, children (yes/no), employment status, relationship status, and living arrangement. Educational level was dichotomized into lower/higher. Lower education was defined as special education or incomplete compulsory schooling (up to 9 years) as defined by the Swiss Federal Department of Foreign Affairs [36]. Higher education included completion of upper secondary school, technical schooling, or university degree.

Participant self-reported medical diagnoses were categorized using the ICD-11 coding system [33]. Coding was chosen for its internal validity and proven efficacy in previous rare disease research [37]. Participants also self-reported their disease course (stable was being specified as “It is stable; not expected to change very much over time” and unstable as either “It is progressive; expected to get more severe over time,” “It is episodic; there are periods of time when it is stable or get better followed by periods where it gets worse,” “It is improving; it is expected to get better over time,” and “Unknown.”), membership in patient organizations (yes/no), time since diagnosis (years), psychiatric diagnoses (yes/no), and number of misdiagnoses. Those individuals who could not recall the exact number of misdiagnoses were given the option of responding “I do not know” or choosing from pre-defined intervals (1–2, 3–5, 5–10, >10). In cases where participants chose an interval, the median of the interval was used for analysis.

#### Access to Healthcare

To assess healthcare access, we used the Perception of Access to Healthcare Questionnaire (PAHQ), a 27-item instrument with a five-point Likert-type scale from 0 (“not at all”) to 5 (“almost always”) [11]. The PAHQ has recently been adapted for individuals with rare diseases in Switzerland and undergone psychometric evaluation [38]. Given the significance of cost-related barriers in rare diseases, we retained the question (item 19) assessing direct healthcare costs. The PAHQ comprises six subscales: availability (e.g., “The facilities of the health center meet the health needs of the clients”; α = 0.70), accessibility (e.g., “The time required to reach the health center is appropriate”; α = 0.92), affordability (e.g., “Cost is a serious barrier to using healthcare”; α = 0.48), adequacy (e.g., “The working hours of the public health center are suitable for receiving services from these centers”; α = 0.80), acceptability (e.g., “The quality of services provided in the health center is acceptable”; α = 0.92), and awareness (e.g., “Health workers try to make sure I fully understand the health information provided”; α = 0.87).

#### Health-Related Quality of Life

The Short Form Health Survey (SF-12) is a validated 12-item questionnaire designed to assess various health dimensions, including physical functioning, role limitations due to physical health, bodily pain, general health, vitality, social functioning, role limitations due to emotional problems, and mental health [39]. It aggregates these into two main scores: the Mental Component Summary (MCS) (α = 0.88) and the Physical Component Summary (PCS) (α = 0.87). Higher scores on the SF-12 indicate better HRQoL.

### Statistical Analysis

Analysis was conducted using R statistical software [40]. We applied the PAM to define clusters of healthcare access based on the PAHQ subscale means [41]. Briefly, PAM robustly considers outliers and uses actual data points as medoids to represent clusters [42]. Medoids are the most centrally located objects within a cluster, acting as a reference point around which other objects are grouped. The algorithm iteratively assigns each observation to the nearest median and minimizes within-cluster dissimilarity, enhancing cluster homogeneity. Determining the optimal number of clusters involves the elbow test, though its interpretative challenges necessitate the average silhouette width (ASW) measure for better accuracy [43]. ASW assesses the correctness of the assignment of data objects to clusters by considering both the separation between clusters and the cohesion within clusters [42]. Consistency of clusters was verified using bootstrapping (100 draws) to calculate the average Jaccard similarity index, with values above 0.75 indicating consistency [44, 45]. Additionally, agglomerative hierarchical clustering with “complete” and “ward” linkage methods was performed for further validation [46].

Subsequent analyses included multiple logistic regression to explore relationships between associated individual characteristics and cluster membership for each PAHQ subscale. Variable selection was conducted through elastic net regression using the Caret package, effectively handling multicollinearity and overfitting [47]. Elastic net, a combination of lasso and ridge regression, steers the coefficient estimates towards zero for regularization, mitigating overfitting [48]. The process involved five-fold cross-validated elastic net regression to identify variables associated with cluster membership. This was followed by logistic regression, reporting odds ratios for the variables’ impact on cluster membership. To assess the associations between access profiles and HRQoL, multiple regression analysis was conducted using the lower access cluster as the primary predictor, along with the individual characteristics that were identified in the previous logistics regression as being significantly associated with access. The magnitude of odds ratios was interpreted as small if the odds ratio was 1.68 or less, medium if it was between 1.68 and 3.47, and large if it was 6.71 or more [49]. All continuous variables were z-standardized to ensure that effect sizes could be compared. In line with the exploratory nature of this study, we did not adjust for multiple testing to avoid decreasing sensitivity to true effects, thereby minimizing Type II errors [50]. Everything *p* < 0.05 was considered significant.

## Results

### Participant Characteristics

Participant characteristics (*N* = 341) are reported descriptively in Table 1. On average, participants were 48 ± 15 years. Forty percent of participants identified as male, 57% had a high level of education, and 40% reported a stable disease course. The most prevalent diseases, per ICD-11 classification, included developmental anomalies, diseases of the nervous system, and endocrine, nutritional, or metabolic diseases.

**TABLE 1:** Participant and scale descriptives of full sample (*N* = 341) (Zurich, Switzerland, 2024).

Participant descriptives	
Age, *M (SD)*	47.58 (15.36)
Male gender, *n* (%)	135 (40%)
Higher education, *n* (%)	194 (57%)
Swiss nationality, *n* (%)	316 (93%)
Private insurance, *n* (%)	71 (21%)
Employed, *n* (%)	185 (54%)
Living alone, *n* (%)	84 (25%)
Single, *n* (%)	220 (65%)
No children, *n* (%)	151 (44%)
Time since diagnosis (years), M (*SD*)	21.65 (18.38)
Unknown	14
Stable disease course, *n* (%)	135 (40%)
Number of misdiagnoses, *M* (*SD*)	1.62 (2.61)
Not reported by participant	76
Psychiatric diagnosis, *n* (%)	101 (30%)
Disease type according to ICD-11, *n* (%)	
Developmental anomalies	64 (18.8%)
Diseases of the blood or blood-forming organs	29 (8.5%)
Diseases of the circulatory system	8 (2.3%)
Diseases of the digestive system	33 (9.7%)
Diseases of the genitourinary system	1 (0.3%)
Diseases of the immune system	13 (3.8%)
Diseases of the musculoskeletal system or connective tissue	1 (0.3%)
Diseases of the nervous system	64 (18.8%)
Diseases of the respiratory system	8 (2.3%)
Diseases of the skin	2 (0.6%)
Diseases of the visual system	28 (8.2%)
Endocrine, nutritional, or metabolic diseases	63 (18.5%)
Neoplasms	5 (1.5%)
Not categorized in ICD-11	22 (6.5%)
Scale descriptives	
PAHQ, *M* (*SD*)	
Acceptability	3.76 (0.82)
Accessibility	3.72 (1.04)
Adequacy	3.51 (0.75)
Affordability	3.50 (0.82)
Availability	3.66 (0.83)
Awareness	3.68 (0.82)
SF-12, M (*SD*)	
Physical component summary	61.88 (29.68)
Mental component summary	59.88 (18.89)

Note. PAHQ, Perceived access to healthcare questionnaire; SF-12, Short-form 12; *M*, mean; *N*, sample size; *SD*, standard deviation.

### Clustering Analyses of Healthcare Access

We computed clusters using different variations: all items, the six PAHQ subscales, and a combination of the accessibility and availability subscales, as previously done by Tian et al. [23]. The highest ASW for the five-subscale approach was 0.37 for two clusters (i.e., lower access and higher access) (Supplementary Figure S5). Clusters were clearly separated (Supplementary Figure S6) with distinct silhouettes for the two-cluster solution (Supplementary Figure S7). The mean Jaccard value of 0.99 indicates high stability of identified clusters and agglomerative hierarchical clustering confirmed that two clusters were optimal (Supplementary Figures S8, S9).

**FIGURE 1:**
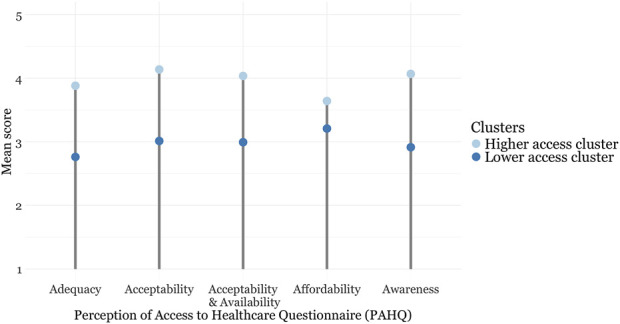
Subscale scores for the Perception of Access to Healthcare Questionnaire stratified by cluster (Zurich, Switzerland, 2024).

The lower access cluster was smaller (*n* = 114) and had lower mean scores across all PAHQ subscales (Table 2) (see Figure 1). The higher access cluster comprised 227 individuals. Descriptive statistics for socio-demographic and disease-related factors, as well as SF-12 scores, are detailed in Supplementary Table S10.

**TABLE 2:** Associated individual characteristics of the lower healthcare access cluster using a 2-class logistic regression model (Zurich, Switzerland, 2024).

Effect	OR	B	*SE*	Wald	95% CI		*p*
					*LL*	*UU*	
Age (z-Standardized)	0.79	−0.236	0.156	2.265	0.582	1.072	0.132
Male gender	1.49	0.396	0.303	1.706	0.820	2.691	0.192
Lower education	0.80	−0.223	0.295	0.573	0.449	1.426	0.449
Foreign nationality	2.25	0.809	0.648	1.560	0.631	7.997	0.212
Single	0.68	−0.390	0.303	1.659	0.374	1.226	0.198
Stable disease course	1.93	0.659	0.313	4.431	1.047	3.570	0.035
Number of misdiagnoses (z-Standardized)	1.41	0.343	0.142	5.856	1.067	1.861	0.016
Psychiatric diagnosis	1.70	0.532	0.310	2.940	0.927	3.126	0.086
Disease type							
Developmental anomalies	1.40	0.337	0.361	0.875	0.690	2.842	0.350
Diseases of the immune system	0.15	−1.921	1.094	3.084	0.017	1.250	0.079
Diseases of the nervous system	3.34	1.205	0.415	8.435	1.479	7.526	0.004
Endocrine, nutritional, or metabolic diseases	2.20	0.789	0.565	1.946	0.727	6.662	0.163

Note. *N* = 341. B, unstandardized regression coefficient; OR, odds ratio; CI, confidence interval; *LL*, lower limit; *UL*, upper limit. Wald = wald test statistic. Statistical significance was determined at *p* < 0.05. The variables listed denote the reference categories used for comparison in the regression analysis. Estimates are provided for other categories relative to these reference categories.

### Associations With Individual Characteristics of Lower Healthcare Access Clusters and Health-Related Quality of Life

Regression analysis revealed an unstable disease course, a higher number of misdiagnoses, and a disease of the nervous system were associated with higher odds of belonging to the lower access cluster (Table 2). These odds ratios were all of small magnitude.

Participants in the lower access cluster scored significantly lower on both physical and mental component scales compared to those in the higher access cluster (Table 3).

**TABLE 3:** Regression results of the association between the lower access cluster and the Short Form 12 Health Survey physical and mental component scores (Zurich, Switzerland, 2024).

Predictor	β	*b*	*b* 95% CI [LL, UL]	*p*	Fit
Physical component score					
Higher healthcare access class	−0.144	−8.960	[−16.140, −1.780]	0.015	
Stable disease course	0.329	19.529	[12.744, 26.313]	0.000	
Number of misdiagnoses (z-standardized)	−0.223	−6.477	[−9.834, −3.120]	0.000	
Diseases of the nervous system	−0.134	−10.648	[−19.575, −1.722]	0.020	
					*R* ^*2*^_*adj.*_ = 0.264
					*p* = 0.000
					*F(4, 231) = 25.09*
Mental component score					
Higher healthcare access class	−0.126	−5.202	[−10.606, 0.202]	0.059	
Stable disease course	−0.130	−5.125	[−10.231, −0.019]	0.049	
Number of misdiagnoses (z-standardized)	−0.139	−2.681	[−5.207, −0.154]	0.038	
Diseases of the nervous system	0.039	2.040	[−4.679, 8.758]	0.550	
					*R* ^*2*^_*adj.*_ = 0.056 *p* = 0.002
					*F(4, 231) = 18.88*

Note. *N* = 341. β, standardized regression coefficient; *b*, unstandardized regression coefficient; CI, confidence interval; *LL*, lower limit; *UL*, upper limit; *R* ^*2*^_*adj.*_, proportion of variance explained, adjusted; *F*, F-statistic; *p*, *p*-value. Statistical significance was determined at *p* < 0.05. The variables listed denote the reference categories used for comparison in the regression analysis. Estimates are provided for other categories relative to these reference categories.

## Discussion

This study aimed to enhance our understanding of healthcare access disparities among adults with rare diseases. We had three main objectives. First, using unsupervised PAM, we categorized clusters within each dimension of healthcare access, identifying two distinct clusters based on the PAHQ mean scales (lower and higher access, respectively). Second, we explored individual characteristics associated with the lower access cluster, identifying that an unstable disease course, a higher number of misdiagnoses, and a disease of the nervous system increased the odds. Third, we examined how membership in the lower access cluster affected HRQoL, as assessed by SF-12 scores. Findings indicate that the lower access cluster had significantly lower physical and mental component scores compared to the higher access cluster.

### Clusters of Healthcare Access

Using PAM, we categorized healthcare access into two distinct clusters based on the PAHQ mean subscales. This aligns with previous findings that identified one cluster with consistently higher scores across all dimensions of access and no access difficulties, and another cluster with difficulties in one or more access dimensions in studies focusing on healthy adults [22, 23]. Our findings underscore the heterogeneity of rare diseases and the varying impact on healthcare access. Two PAHQ dimensions (adequacy and awareness) scored particularly low in the lower access cluster.

In relation to the adequacy dimension, multidisciplinary care required for chronic diseases frequently exceeds the capacity of Swiss providers [51]. Patients find it challenging to find specialists and coordinate care, which is further complicated by long wait times [52]. In 2017, the Swiss healthcare system established a coordination platform named kosek with nine specialist centers for rare diseases. Additionally, patients with an established rare disease diagnosis can receive care through disease-specific reference centers [53]. Future research should explore the patient experience with specialized centers to better understand if and how such services support patient care.

Regarding the awareness dimension, findings indicate the lack of understandable information (i.e., lay language) remains a barrier for patients. Patient knowledge deficits are exacerbated by general practitioners who often have limited awareness and understanding of rare diseases and who are unaware of resources providing reliable rare disease information [54, 55]. Moreover, both medical jargon and limited health literacy/numeracy complicate shared decision-making [56]. Patients frequently resort to doing their own research to learn about their condition which presents potential for finding inaccurate information [57].

Consequently, the improving the integration and coordination of specialized care and facilitating access to accurate, understandable information remain critical areas for addressing the unique challenges faced by patients with rare diseases.

### Associations Between Cluster Membership, Individual Characteristics, and Health-Related Quality of Life

We identified several variables associated with belonging to the lower access cluster including an unstable disease course, higher number of misdiagnoses, and nervous system conditions. Previous research indicates that unstable diseases are correlated with an increased likelihood of requiring healthcare support and a higher frequency of healthcare interactions [19]. Frequent healthcare utilization is a driver of increased healthcare costs that can negatively affect accessible services, especially for individuals with reduced mobility [58, 59]. Unstable disease trajectories are also associated with comorbid mental health problems [30]. Mental health issues make access to care more difficult as patients have to simultaneously manage both physical and mental healthcare [60]. An unstable disease course complicates disease management for healthcare providers and patients alike. Cumulatively, these complexities highlight the need for interventions to extend the reach of care especially for individuals affected by a condition with an unstable disease course.

A greater number of misdiagnoses was associated with higher odds of belonging to the lower access cluster. This is consistent with previous findings showing that misdiagnosis contributes to timely correct diagnosis and initiation of appropriate treatment(s). Incorrect diagnoses contribute to patients feeling they are not believed or taken seriously by healthcare professionals [61]. Such experiences erode patient confidence and trust in both healthcare providers and health systems [62]. Ultimately, lingering effects of misdiagnosis can exacerbate inequalities in access to care. Patients may disengage and withdraw from the system posing additional barriers to accurate diagnosis and effective treatment [14]. There is a need for healthcare systems to improve diagnostic accuracy, enhance effective communication, and rebuild and maintain trust with patients.

We found rare diseases affecting the nervous system were associated with reduced access to healthcare. Such disorders are frequently associated with limited mobility and cognitive impairments that further hamper access [28, 63]. In parallel, lack of awareness and use of mobility-enhancing devices may further compound problems of access [64]. Recently, the COVID-19 pandemic has exacerbated the situation by limiting access to rehabilitation and hospital services, resulting in a decline in motor and non-motor function in patients with neurologic conditions [65]. A deeper understanding of specific barriers is needed to develop and implement interventions that enhance access to care for those affected by neurologic conditions.

Consequently, the development of targeted strategies that address the multifaceted barriers to diagnosis and treatment is essential. These strategies must not only enhance diagnostic accuracy and patient-provider trust but also consider the unique challenges posed by an unstable disease course and nervous system conditions.

Membership in the Lower access cluster was significantly associated with diminished HRQoL scores for both physical and mental scales. These observations support the notion that reduced access to healthcare correlates with poorer health outcomes [28]. However, the dynamics between healthcare access and health outcomes are not fully understood. Although our findings align with existing literature linking access and outcomes [66], our results should be interpreted cautiously given the cross-sectional design. Decreased HRQoL is associated with reduced access, yet may be an outcome and/or a predictor of decreased access [19, 60]. Thus, further research is needed to clarify the observed relationships.

### Practical Implications

Observed low scores in the awareness dimension point to the need for creating lay language patient-facing information. Zeltner et al. [67], created understandable educational materials on inborn errors of metabolism and its treatment that significantly increased patient knowledge. Others have partnered with patients to co-create patient-friendly materials for congenital hypogonadotropic hypogonadism. Such co-created materials score highly on understandability and actionability using the “gold standard” Patient Education Materials Assessment Tool [68, 69]. Indeed, co-creation can be effective as it centers on patient priorities and preferences [70]. Thus, development of patient education materials offers a promising avenue for improving disease- and treatment-related knowledge and possibly enhancing healthcare access. A recent framework for developing patient education materials specific to rare diseases could guide future interventions [71].

Individuals with an unstable disease course and neurologic disorders are at risk for lower access to care. These observations are likely due to mobility constraints and/or struggles of coordinating care for multiple providers. Telehealth represents an avenue for improving access for such patient populations. Telehealth has been shown to be beneficial in chronic disease management enabling access care for individuals with reduced mobility [72, 73]. The benefits of telemedicine extend beyond transportation barriers, as telemedicine also contributes to improved healthcare efficiency and reduced healthcare costs. Thus, telehealth can reduce waiting times and does not depend on facility structures such as wheelchair access thereby addressing the adequacy dimension [73].

In summary, strategic development of easy-to-understand educational materials and the expansion of telemedicine services are critical areas for addressing some of the multiple challenges faced by rare disease patients.

### Strengths and Limitations

A relative strength of this study is its grounding in robust theoretical frameworks. To date, most rare disease research examining access has lacked a theoretical underpinning. We used validated, theory-based instruments, like the PHAQ [11], to comprehensively analyze healthcare access dimensions and their associated individual characteristics. This work also draws on theories relating to sociodemographic and disease-specific factors of access as proposed by Andersen et al. [21]. Such determinants have been overlooked in the literature on rare diseases as much of the research to date has primarily focused on describing the state of access but not its associated individual characteristics [16, 30, 59]. As such, our approach supports the validity and replicability of our findings [74] and provides a detailed overview of healthcare access for Swiss rare disease patients. Another relative strength of the work is the analytic approach employed. Using the PAM algorithm is an innovation in rare disease research and was effective for classifying healthcare access and handling outliers present in rare disease patient populations [42]. The strategy can inform tailored interventions responding to the unique healthcare needs of people with rare diseases and aligns with tenets of precision healthcare [75]. The analyses were only possible due to the relatively large sample size in the present study that included heterogeneous rare disease entities. Previous research has shown that rare diseases in general share many common hurdles, especially with regard to healthcare access and utilization [14, 76].

It is important to note that the cross-sectional design of our study limits the validation of clusters against health outcomes such as HRQoL and does not allow for the analysis of temporal relationships, such as the impact of aging, the timing of misdiagnoses or medical interventions, nor changes in the disease course on healthcare access and outcomes. While our analysis provides insights into patterns of healthcare access and associated characteristics among individuals with rare diseases, it cannot determine temporal sequences, precluding causal inferences. Additionally, the simplicity of the models used may overlook critical nuances in real-world access, which involves multifaceted interactions among social, cultural, economic, and systemic factors. Our analysis did not explore these complex interactions or interaction effects between individual characteristics, such as how the influence of one factor (e.g., education or gender) on healthcare access might depend on another factor (e.g., age, disease severity, or economic status). Future research employing a longitudinal approach would be essential to capture the dynamic relationships between healthcare access, misdiagnoses, disease progression, and health outcomes. Such studies could also identify critical periods where interventions might be most effective and explore how interactions between variables—including social, cultural, economic, and systemic factors—affect these relationships, particularly in understanding how combinations of factors like age and timing of intervention influence the stability of the disease course and access to medical care.

While our study provides valuable insights into healthcare access among individuals with rare diseases, it is important to acknowledge that not all dimensions of access carry equal weight in real-world scenarios. The framework used in our analysis treats all dimensions as equally important. However, in practice, certain dimensions may be more critical depending on the context. For instance, availability might become a paramount concern during emergencies where immediate access to treatment is essential. To better account for these varying contexts and situations, more naturalistic study designs, such as daily diary studies or experience sampling methodologies, may be better suited to capture the variability of access dimensions and their varying importance across different situations.

The study used PAM to create access-to-care clusters due to the lack of existing cutoffs. The term “lower” rather than “low” was chosen to emphasize the relative comparison to the “higher” access cluster. While it is unclear if this “lower access” is clinically significant or comparable across other contexts, the findings show that those in the lower access cluster face more severe HRQoL impairments. However, given the cross-sectional design of our study, we must be cautious in interpreting these findings as evidence of a direct causal impact. While the association between reduced access to healthcare and poorer HRQoL is present, the study design does not allow us to establish temporal sequences or causality. It is possible that lower HRQoL could also contribute to reduced access to healthcare, creating a bidirectional relationship. Thus, in addition, longitudinal studies could help elucidate the clinical significance of the identified access clusters and establish benchmarks (such as PAHQ thresholds) as metrics of care.

### Conclusion

Findings advance our understanding of disparities in healthcare access among adults with rare diseases by identifying distinct access clusters, their associated individual characteristics and exploring their association with HRQoL. These insights are crucial for developing person-centered interventions tailored to the individual needs of this unique patient population. Our results inform the development of targeted interventions to enhance efficacy and improve patient outcomes. Results also highlight the need for improved resource allocation in relation to care coordination and patient education/communication. This research lays the groundwork for further studies and policy initiatives aimed at creating more equitable and effective healthcare systems globally.
