# Supplementary material for: Lower Healthcare Access and Its Association With Individual Factors and Health-Related Quality of Life in Adults With Rare Diseases in Switzerland
Source: Int J Public Health. 2024 Sep 25;69:1607548. doi: 10.3389/ijph.2024.1607548 (PMC11461209; doi:10.3389/ijph.2024.1607548)
Supplement: Supplementary file 1 [file DataSheet1.docx]

**Supplementary Table S1.** Sensitivity analysis.

| PAHQ subscales | No missing SF-12 data (*N* = 302) | | Missing SF-12 data (*N* = 41) | |  |  |  |
| --- | --- | --- | --- | --- | --- | --- | --- |
|  | *M* | *SD* | *M* | *SD* | *U* | *p* | *δ* |
| Acceptability | 3.76 | 0.83 | 3.78 | 0.77 | 6192.5 | 0.999 | 0.000 |
| Accessibility | 3.72 | 1.04 | 3.68 | 1.00 | 6262.5 | 0.904 | 0.012 |
| Adequacy | 3.50 | 0.77 | 3.58 | 0.61 | 5990.5 | 0.736 | -0.032 |
| Affordability | 3.52 | 0.80 | 3.29 | 0.96 | 6973.0 | 0.186 | 0.126 |
| Availability | 3.65 | 0.84 | 3.66 | 0.73 | 6372.5 | 0.758 | 0.029 |
| Awareness | 3.69 | 0.83 | 3.62 | 0.79 | 6685.0 | 0.405 | 0.080 |

**Note.** *M* = mean. *N* = sample size. *SD* = standard deviation. *U* = U-statistic. *p* = p-value. PAHQ = Perceived Access to Healthcare Questionnaire. *δ =* Cliff’s Delta*.*

**Supplementary Figure S2.** Boxplot of Perceived Access to Healthcare Questionnaire subscales

**
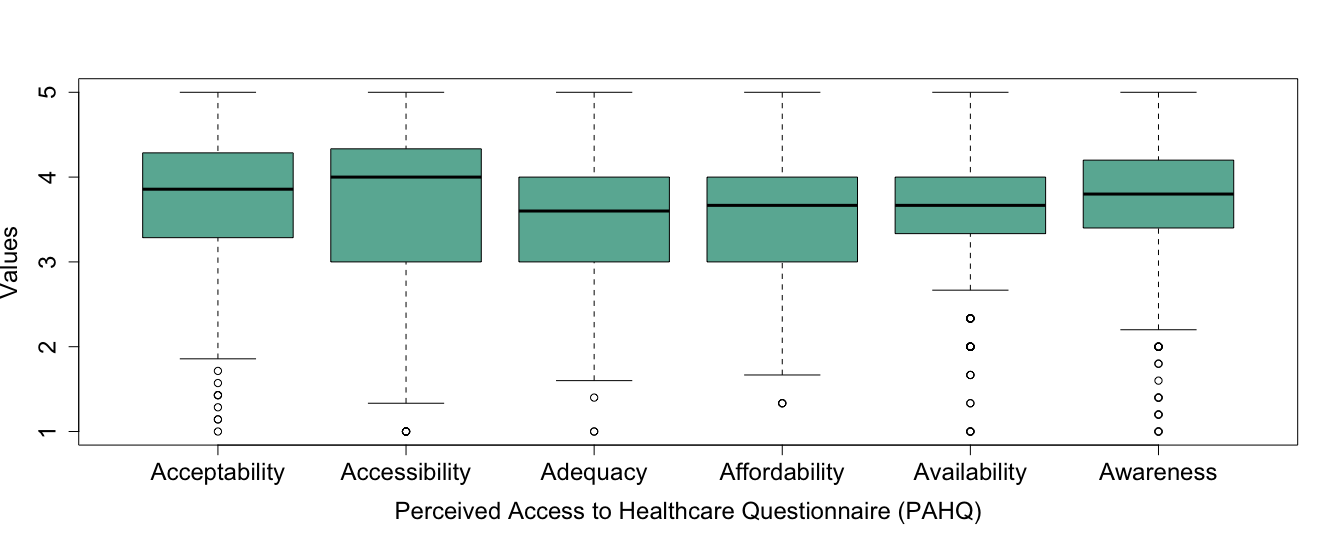
**

**Supplementary Figure S3.** Boxplot of Short Form 12 Health Survey subscales

**
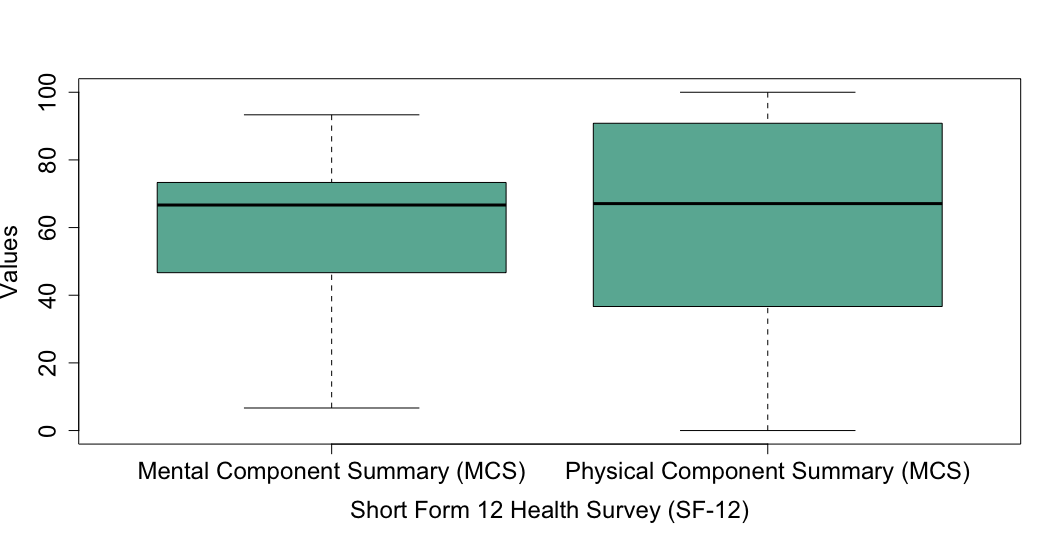
**

**Supplementary Table S4.** Overview across removed disease types

| Disease type, *n* = 28 | |  |
| --- | --- | --- |
| Disease types below 10 cases according to ICD-11 categorization | | |
| Certain infectious or parasitic diseases | 1 |  |
| Diseases of the circulatory system | 8 |  |
| Diseases of the genitourinary system | 1 |  |
| Diseases of the musculoskeletal system or connective tissue | 1 |  |
| Diseases of the respiratory system | 8 |  |
| Diseases of the musculoskeletal and connective tissue | 1 |  |
| Diseases of the skin | 2 |  |
| Neoplasms | 5 |  |

**Supplementary Table S5.** Silhouette scores of Perceived Access to Healthcare Questionnaire subscales.

| Average Silhouette Scores PAHQ subscales (*N* = 341) | | | |
| --- | --- | --- | --- |
| Model | 5- Subscales | 6-Subscales | Item-level |
| 2-clusters | 0.37 | 0.34 | 0.11 |
| 3-clusters | 0.29 | 0.24 | 0.20 |
| 4-clusters | 0.30 | 0.26 | 0.13 |

**Notes.** PAHQ = Perceived Access to Healthcare Questionnaire.

**Supplementary Figure S6.** Partition around medoids clustering results.


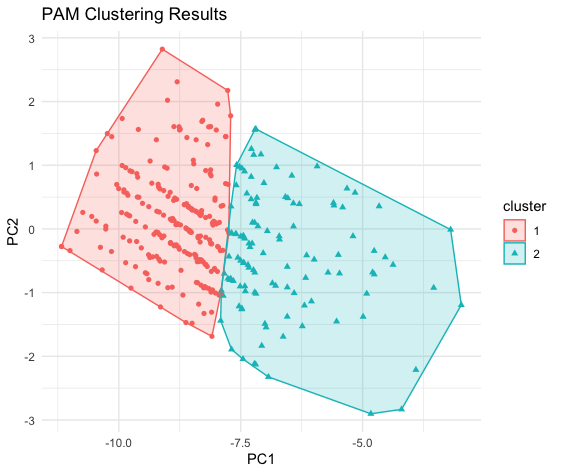


**Supplementary Figure S7.** Silhouette plot of Perceived Access to Healthcare Questionnaire subscales.

**
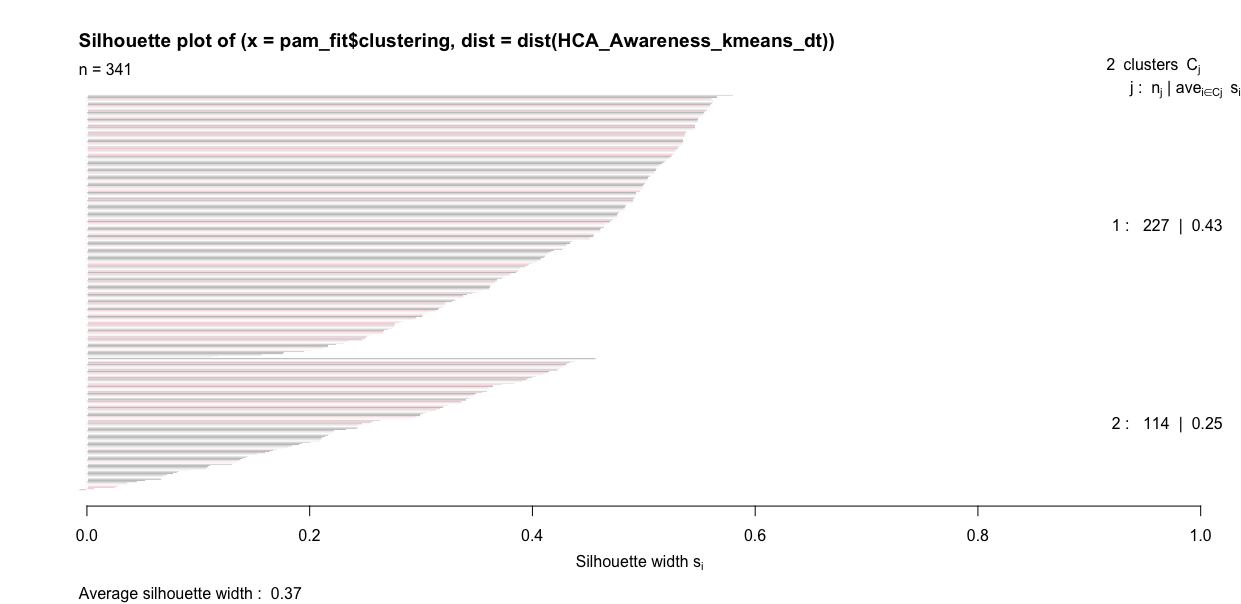
**

**Supplementary Figure S8.** Hierarchical clustering with complete linkage.

**
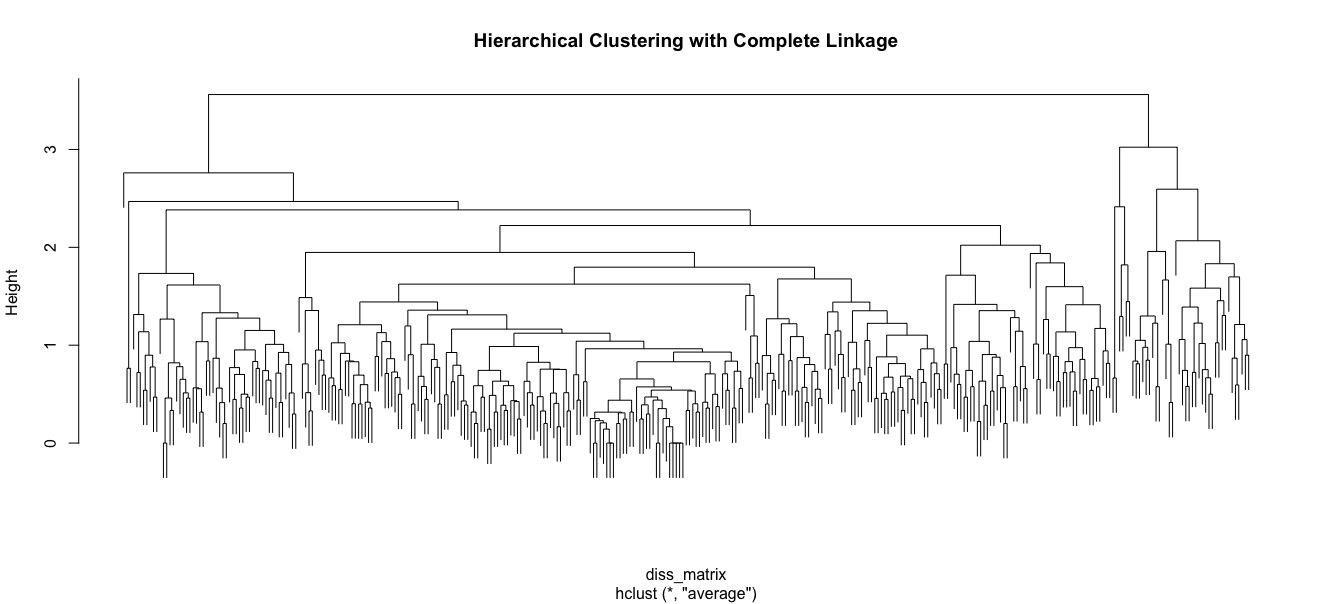
**

**Supplementary Figure S9.** Hierarchical clustering with ward linkage.

**
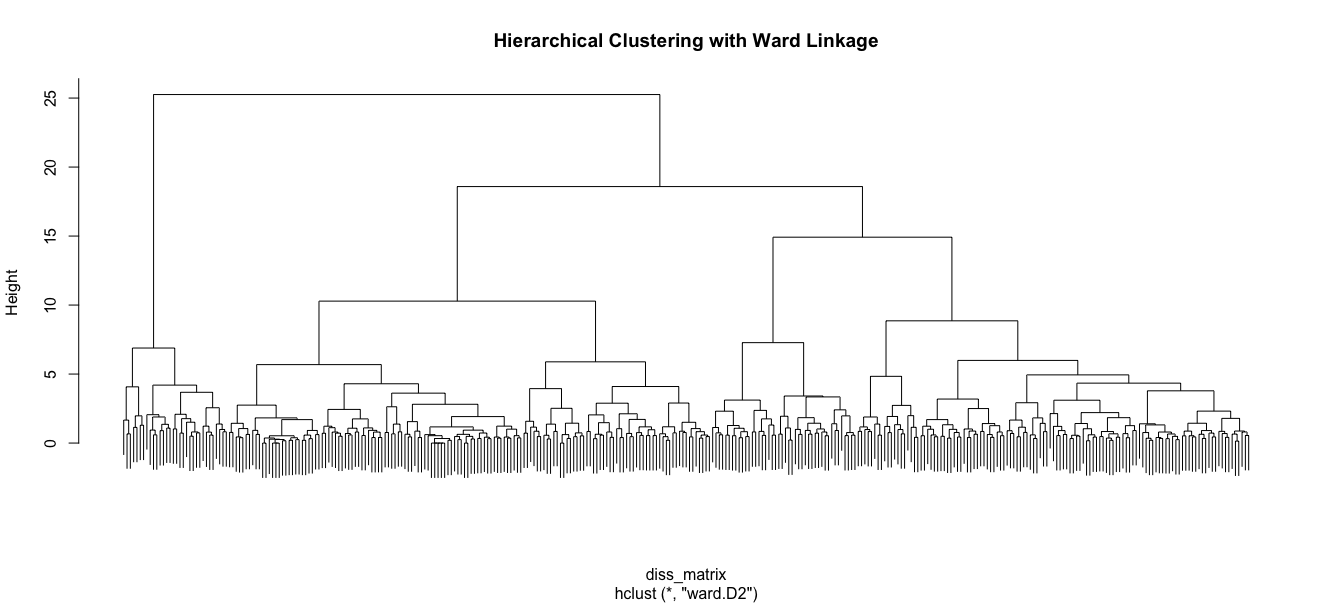
**

**Supplementary Table S10.** Descriptive statistics of full sample divided by cluster.

|  | | | ***Lower healthcare access cluster***  ***N* = 114** | ***Higher healthcare access cluster***  ***N* = 227** |
| --- | --- | --- | --- | --- |
| Age, *M* (*SD*) | | | 46 (14) | 48 (16) |
| Gender male, *n* (%) | | | 40 (35%) | 95 (42%) |
| Education high ^a^, *n* (%) | | | 65 (57%) | 129 (57%) |
| Swiss nationality, *n* (%) | | | 106 (93%) | 210 (93%) |
| Insurance general, *n* (%) | | | 92 (81%) | 178 (78%) |
| Employed, *n* (%) | | | 59 (52%) | 126 (56%) |
| Living alone, *n* (%) | | | 33 (29%) | 51 (22%) |
| Single, *n* (%) | | | 46 (40%) | 75 (33%) |
| Does not have children, *n* (%) | | | 66 (58%) | 124 (55%) |
| Time since diagnosis (years), *M* (*SD*) | | | 18 (18) | 23 (18) |
| Unknown | | 5 | 9 |  |
| Stable disease course, *n* (%) | | | 29 (25%) | 106 (47%) |
| Number of misdiagnoses, *M* (*SD*) | | | 2.40 (2.99) | 1.24 (2.31) |
| Unknown | | 26 | 50 |  |
| Psychiatric diagnosis, *n* (%) | | | 48 (42%) | 53 (23%) |
| Disease type according to ICD-11, *n* (%) | | |  |  |
| Developmental anomalies | 22 (19%) | 42 (19%) |  |  |
| Diseases of the blood or blood-forming organs | 9 (7.9%) | 20 (8.8%) |  |  |
| Diseases of the digestive system | 8 (7.0%) | 25 (11%) |  |  |
| Diseases of the immune system | 1 (0.9%) | 12 (5.3%) |  |  |
| Diseases of the nervous system | 30 (26%) | 34 (15%) |  |  |
| Diseases of the visual system | 13 (11%) | 15 (6.6%) |  |  |
| Endocrine, nutritional, or metabolic diseases | 19 (17%) | 44 (19%) |  |  |
| Not categorized in ICD-11 | 6 (5.3%) | 16 (7.0%) |  |  |
| PAHQ, *M* (*SD*) | | |  |  |
| Acceptability | 3.01 (0.79) | 4.14 (0.53) |  |  |
| Accessibility & Availability | 3.05 (1.04) | 4.05 (0.86) |  |  |
| Adequacy | 2.76 (0.57) | 3.88 (0.52) |  |  |
| Affordability | 3.21 (0.89) | 3.64 (0.75) |  |  |
| Awareness | 2.94 (0.77) | 4.02 (0.59) |  |  |
| SF-12, *M* (*SD*) | | |  |  |
| Mental component summary | 55 (21) | 62 (17) |  |  |
| Physical component summary | 50 (30) | 68 (28) |  |  |
| *Note.* PAHQ = Perceived access to healthcare questionnaire. SF-12 = Short-form 12*. M* = mean. *N* = sample size. *SD* = standard deviation. | | | |  |

^a^ 0 = special education school / not completed mandatory school education, completion of mandatory schooling (9 years), 1 = higher degree upper secondary school / secondary school & A-levels / technical school / seminar / university of applied sciences & University / ETH.
